# Reporting of a surveillance system of multidrug-resistant organisms in an Italian hospital

**DOI:** 10.1186/1753-6561-5-S6-P298

**Published:** 2011-06-29

**Authors:** MM D'Errico, A Marigliano, I Pellegrini, MG Gioia, S Savini, M Gigli, E Martini, E Manso, P Barbadoro

**Affiliations:** 1 Polytechnic University of The Marches, Ancona, Italy; 2 Hospital Hygiene Service, AOU Ospedali Riuniti, Ancona, Italy; 3 AOU Ospedali Riuniti, Hospital Hygiene Service, Ancona, Italy; 4 Hospital Hygiene Service, Ancona, Italy; 5 Microbiology Laboratory, AOU Ospedali Riuniti, Ancona, Italy

## Introduction / objectives

Healthcare facilities are monitoring Multidrug-Resistant Organisms (MDROs) because of their increasing incidence. A surveillance system for MDROs isolated from routine clinical cultures was set up in a teaching hospital in Central Italy.

## Methods

Since January 2009, daily, Hospital Hygiene Service personnel collects MDROs microbiological data. Data are automatically entered in a software, continuously updated.Only the first MDRO isolate recovered from a patient is considered. The monthly report includes: newly recovered MDRO isolates; incidence rate of MDRO infection/colonization (No. of first MDRO isolates per patient for each unit/1000 patient days);statistical process control charts.

## Results

From 01/01/2009 to 31/12/2010, 1160 MDROs were isolated; the most represented MDRO was ESBL *E.coli* (n=216; 18.6%) followed by MRSA (n=178; 15.3%) and *A.baumannii* (n=158; 13.6%). 26% (n=302) of the MDROs isolated came from Intensive Care Units (ICUs). The mean MDROs incidence rate was 1.96 and 10.68/1000 patient days when considering the whole hospital and the ICUs, respectively. Analyzing the rates over time, an extremely fluctuating trend was observed: the ICUs MDROs rate varied from a minimum of 4.73 to a maximum of 20.88. No outbreaks were documented and the warning and control limits were never been exceeded. Among the possible factors contributing to this phenomenon, the sampling rate (No. of all samples sent to the microbiology laboratory/patient days) was analyzed, but no significant changes over time were found.

## Conclusion

Our data highlighted the changeable trend of MDROs rates; a more accurate study is ongoing to assess what kind of variables can influence the spread of MDROs (staff? mini-clusters?) in order to provide the best preventive strategies.

## Disclosure of interest

None declared.

